# Supplementary material for: Artificial intelligence-assisted prostate cancer diagnosis for reduced use of immunohistochemistry
Source: Commun Med (Lond). 2025 Oct 15;5:425. doi: 10.1038/s43856-025-01185-y (PMC12528698; doi:10.1038/s43856-025-01185-y)
Supplement: Supplementary file 2 — Supplemental material [file 43856_2025_1185_MOESM2_ESM.pdf]

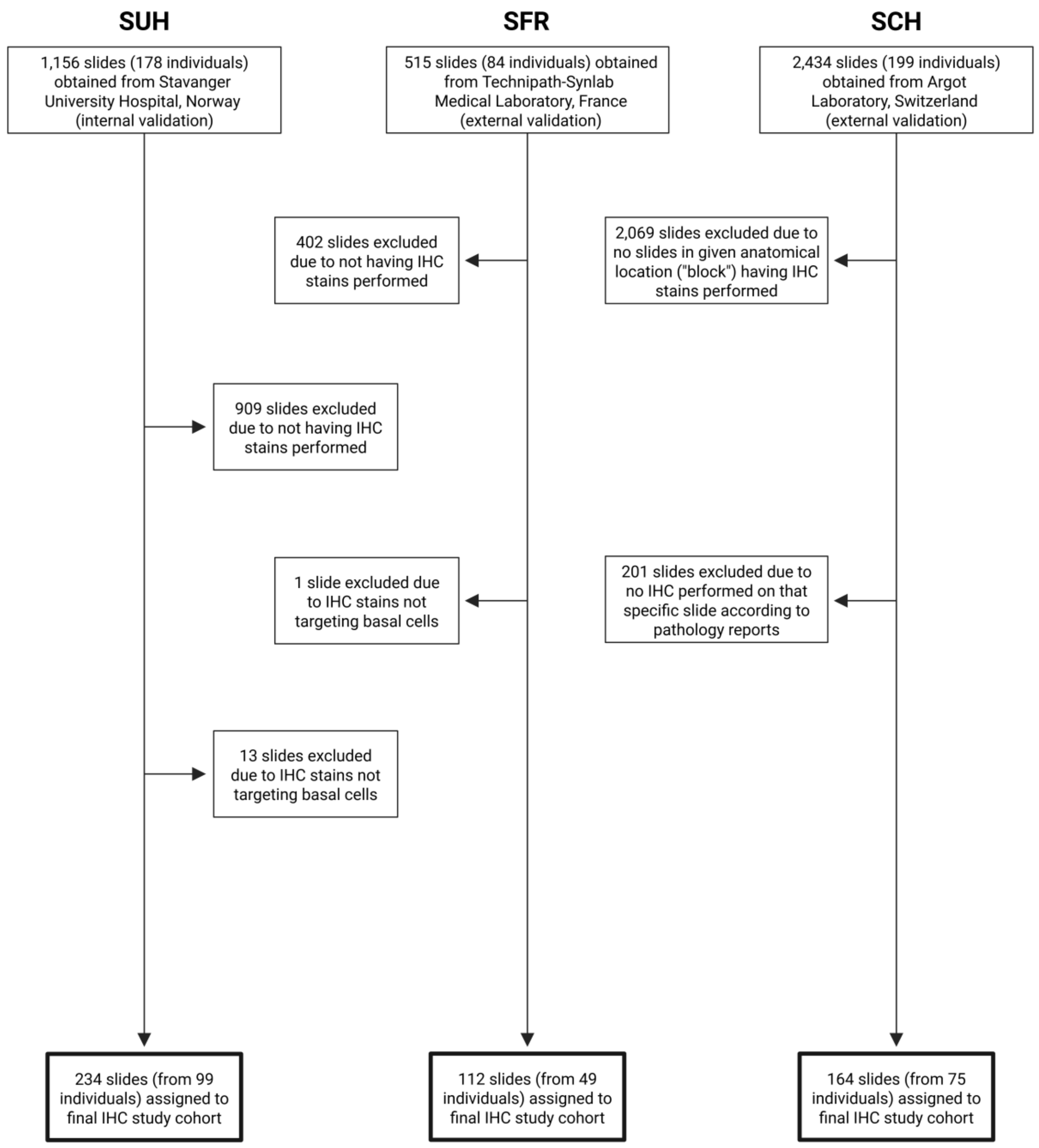

Supplementary Figure 1: CONSORT diagram for the SUH, SFR and SCH cohorts. IHC=immunohistochemistry, SCH=Synlab Laboratory Switzerland, SFR=Synlab Laboratory France, SUH=Stavanger University Hospital.

	Cohorts	SUH (n = 234)			SFR (n = 112)			SCH (n = 164)		
	**AI model**	**TS**	**UFM**	**VFM**	**TS**	**UFM**	**VFM**	**TS**	**UFM**	**VFM**
	**AUROC**	**0.980**	**0.962**	**0.974**	**0.993**	**0.966**	**0.990**	**0.951**	**0.951**	**0.963**
**Threshold 0.50**	**Sensitivity**	0.914	0.943	0.971	0.935	0.957	0.978	0.899	0.909	0.919
	**Specificity**	0.930	0.876	0.892	0.955	0.803	0.894	0.831	0.846	0.846
	**True positives (TP)**	96	99	102	43	44	45	89	90	91
	**False positives (FP)**	9	16	14	3	13	7	11	10	10
	**True negatives (TN)**	120	113	115	63	53	59	54	55	55
	**False negatives (FN)**	9 (8.6%)	6 (5.7%)	3 (2.9%)	3 (6.5%)	2 (4.4%)	1 (2.2%)	10 (10.1%)	9 (9.1%)	8 (8.1%)
	ISUP 1	6	5	3	3	2	1	6*	6*	7*
	ISUP 2	0	0	0	0	0	0	1*	2*	1*
	ISUP 3	0	0	0	0	0	0	3*	1*	0
	ISUP 4	1	0	0	0	0	0	0	0	0
	ISUP 5	2	1	0	0	0	0	0	0	0
	**IHC reduction (TN + FN)**	129 (55.0%)	119 (50.9%)	118 (50.4%)	66 (58.9%)	55 (49.1%)	60 (53.6%)	64 (39.0%)	64 (39.0%)	63 (38.4%)
**Threshold 0.20**	**Sensitivity**	0.952	0.962	0.991	1.000	1.000	0.978	0.929	0.970	0.970
	**Specificity**	0.892	0.799	0.837	0.909	0.439	0.803	0.831	0.569	0.723
	**True positives (TP)**	100	101	104	46	46	45	92	96	96
	**False positives (FP)**	14	26	21	6	37	13	11	28	18
	**True negatives (TN)**	115	103	108	60	29	53	54	37	47
	**False negatives (FN)**	5 (4.8%)	4 (3.8%)	1 (1.0%)	0 (0.0%)	0 (0.0%)	1 (2.2%)	7 (7.1%)	3 (3.0%)	3 (3.0%)
	ISUP 1	2	3	1	0	0	1	3*	1*	3*
	ISUP 2	0	0	0	0	0	0	1*	2*	0
	ISUP 3	0	0	0	0	0	0	3*	0	0
	ISUP 4	1	0	0	0	0	0	0	0	0
	ISUP 5	2	1	0	0	0	0	0	0	0
	**IHC reduction (TN + FN)**	120 (51.3%)	107 (45.7%)	109 (46.6%)	60 (53.6%)	29 (25.9%)	54 (48.2%)	61 (37.2%)	40 (24.4%)	50 (30.5%)
**Threshold 0.10**	**Sensitivity**	0.952	0.971	0.991	1.000	1.000	1.000	0.960	0.980	1.000
	**Specificity**	0.883	0.721	0.767	0.894	0.197	0.712	0.785	0.369	0.692
	**True positives (TP)**	100	102	104	46	46	46	95	97	99
	**False positives (FP)**	15	36	30	7	53	19	14	41	20
	**True negatives (TN)**	114	93	99	59	13	47	51	24	45
	**False negatives (FN)**	5 (4.8%)	3 (2.9%)	1 (1.0%)	0 (0.0%)	0 (0.0%)	0 (0.0%)	4 (4.0%)	2 (2.0%)	0 (0.0%)
	ISUP 1	2	2	1	0	0	0	1*	1*	0
	ISUP 2	0	0	0	0	0	0	0	1*	0
	ISUP 3	0	0	0	0	0	0	3*	0	0
	ISUP 4	1	0	0	0	0	0	0	0	0
	ISUP 5	2	1	0	0	0	0	0	0	0
	**IHC reduction (TN + FN)**	119 (50.9%)	96 (41.0%)	100 (42.7%)	59 (52.7%)	13 (11.6%)	47 (42.0%)	55 (33.5%)	26 (15.9%)	45 (27.4%)
**Threshold 0.01**	**Sensitivity**	1.000	1.000	1.000	1.000	1.000	1.000	1.000	1.000	1.000
	**Specificity**	0.806	0.465	0.504	0.712	0.000	0.167	0.523	0.108	0.339
	**True positives (TP)**	105	105	105	46	46	46	99	99	99
	**False positives (FP)**	25	69	64	19	66	55	31	58	43
	**True negatives (TN)**	104	60	65	47	0	11	34	7	22
	**False negatives (FN)**	0 (0.0%)	0 (0.0%)	0 (0.0%)	0 (0.0%)	0 (0.0%)	0 (0.0%)	0 (0.0%)	0 (0.0%)	0 (0.0%)
	ISUP 1	0	0	0	0	0	0	0	0	0
	ISUP 2	0	0	0	0	0	0	0	0	0
	ISUP 3	0	0	0	0	0	0	0	0	0
	ISUP 4	0	0	0	0	0	0	0	0	0
	ISUP 5	0	0	0	0	0	0	0	0	0
	**IHC reduction (TN + FN)**	104 (44.4%)	60 (25.6%)	65 (27.8%)	47 (42.0%)	0 (0.0%)	11 (9.8%)	34 (20.7%)	7 (4.3%)	22 (13.4%)

Supplementary Table 1: Performance of task-specific and foundation models across sensitivity-prioritized thresholds. AI models' performance across the SUH, SFR, and SCH cohorts under different sensitivity-prioritized thresholds. In a scenario where IHC staining is only requested for AI-predicted positive slides, the reduction in IHC usage corresponds to the total number of negative predictions. False negative predictions indicate missed cancers, and their ISUP distribution is provided. *In the SCH cohort, cancer grading was assigned at the location level (across multiple slides), meaning true grades for individual WSIs are unknown. Instead, we report the overall grade assigned to the location. AI=artificial intelligence, AUROC=area under the receiver operating characteristic curve, IHC=immunohistochemistry, ISUP=International Society of Urological Pathology grade, SCH=Synlab Laboratory Switzerland, SFR=Synlab Laboratory France, SUH=Stavanger University Hospital, TS=task-specific model, UFM=UNI foundation model, VFM=Virchow2 foundation model, WSI=whole-slide image.
